# Conidiobolomycose (entomophthoromycose rhinofaciale) au Gabon, à propos d'une observation

**DOI:** 10.48327/mtsi.v3i4.2023.457

**Published:** 2023-12-13

**Authors:** Stéphanie NTSAME NGOUA, Josaphat IBA BA, Sophie CONIQUET, Ghislaine MOUSSIROU SOUMBOU, Jean Bruno BOGUIKOUMA

**Affiliations:** 1 Service de dermatologie, CHU de Libreville, Libreville, Gabon; 2 Service de médecine interne, CHU de Libreville, Libreville, Gabon

**Keywords:** Conidiobolomycose, Entomophthoromycose rhinofaciale, Itraconazole, Amélioration clinique, La Ngounié, Gabon, Afrique subsaharienne, Conidiobolomycosis, Rhinofacial entomophtoromycosis, Itraconazole, Clinical improvement, Ngounié Province, Gabon, Sub-Saharan Africa

## Abstract

**Introduction:**

La conidiobolomycose ou entomophthoromycose rhinofaciale est une mycose sous-cutanée tropicale rare, réalisant dans les formes évoluées un aspect dysmorphique, typique, du visage en « museau d'hippopotame », dont peu de cas ont été rapportés dans la littérature.

**Méthodologie:**

Nous présentons l'observation d'un patient de 25 ans, vivant en zone équatoriale, au sud du Gabon en environnement forestier humide.

**Résultats:**

Les données histologiques de la biopsie cutanée associées à la présentation clinique étaient compatibles avec le diagnostic de conidiobolomycose. L’évolution initiale était favorable sur le plan esthétique sous itraconazole 300 mg/jour pendant 2 mois et corticothérapie (bolus de méthylprednisone 240 mg/jour pendant 3 jours relayée per os à la dose de 0,5 mg/kg/jour (soit 30 mg/jour) de prednisone), maintenue pendant 3 mois. L'amélioration moyenne nasale n'a pu être complétée par une chirurgie et le malade a été perdu de vue.

**Conclusion:**

Cette deuxième observation de conidiobolomycose au Gabon dans la même province, fait de la Ngounié, un écosystème privilégié de cette affection.

## Abstract (en)

**Background:**

Entomophthoromycosis constitutes a nosological group of subcutaneous mycoses including conidiobolomycosis (rhinofacial form) and basidiobomomycosis (subcutaneous form involving the trunk and the limbs). Conidiobolomycosis is characterized by a progressive nasal and facial deformity giving, in the evolved forms, a “hippopotamus snout”. The literature review finds a hundred cases, with a tropism for the humid tropical regions. Methods. We report the observation of a 25-year-old patient, living in the equatorial zone, in the south of Gabon in a humid forest area, presenting a swollen aspect of the face mainly involving the eyelids, the nose and the upper lips.

**Results:**

The diagnosis of entomophthoromycosis was compatible with the histopathological and clinical aspects. The evolution was favorable in terms of facial aesthetics under itraconazole 300 mg/day for 2 months and corticosteroid therapy (bolus of methylprednisone 240 mg/day for 3 days relayed per os at a dose of 0.5 mg/kg/day, i.e. 30 mg/day) of prednisone), maintained for 3 months. The average nasal improvement could not be completed by surgery and the patient was lost to follow-up.

**Conclusion:**

This second observation of conidiobolomycosis in Gabon in the same province makes Ngounié a privileged ecosystem for this affection.

## Introduction

Les entomophthoromycoses sont des mycoses sous-cutanées tropicales incluant la conidiobolomycose (forme rhinofaciale) et la basidiobomomycose, (forme sous cutanée). Sur le plan évolutif la conidiobolomycose, due à *Conidiobolus coronatus,* réalise une tuméfaction avec déformation du visage aboutissant à un aspect en « museau d'hippopotame » dans les formes évoluées. Il s'agit d'une pathologie rare, dont nous rapportons une observation au Gabon dans le but de rappeler aux praticiens l'existence possible de cas sporadiques dans ce pays et de les sensibiliser au diagnostic.

## Observation

**Figure 1:**
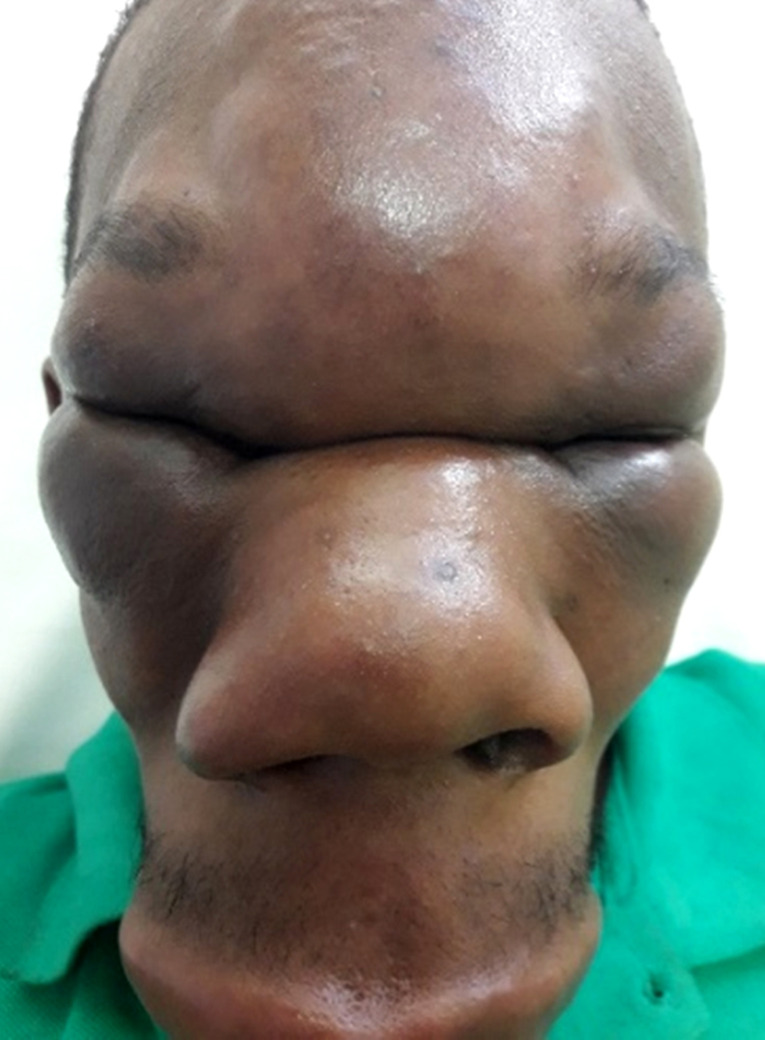
Visage du patient de face Patient's face from the front

Un patient de 25 ans, chauffeur vivant en zone équatoriale à Lébamba (sud du Gabon), dans une zone forestière humide, présentait une tuméfaction indurée du front, des paupières, des pommettes, du nez et de la lèvre supérieure indolore (Fig. 1 et 2), responsable d'une limitation de l'ouverture des yeux et d'une obstruction nasale avec gêne respiratoire au coucher évoluant depuis 3 mois en l'absence de contexte fébrile et d'altération de l’état général. Le reste de l'examen clinique hormis l'existence d'adénopathies cervicales mobiles indolores centimétrique bilatérales, était sans particularité. À la numération formule sanguine, les leucocytes étaient à 10 900 éléments/mm^3^ (60% de neutrophiles, et 5% d’éosinophiles), le taux d'hémoglobine à 10,7 g/dl normochrome normocytaire, et le taux de plaquettes normale. Le taux de C réactive protéine était à 41 mg/l, la glycémie, la fonction rénale et hépatique normales, et les sérologies HIV 1 et 2 négatives. Les radiographies du visage et du thorax étaient normales. L'analyse des 2 fragments de tissu cutané (un fixé, et le second à l’état frais) prélevés, intéressait l’épiderme, le derme, et l'hypoderme. Le derme profond et l'hypoderme était le siège d'une importante réaction granulomateuse lympho-épithéliogigantocellulaire riche en polynucléaires éosinophiles. Les cellules géantes renfermaient des hyphes (filaments mycéliens) rubanés, larges, éosinophiles avec une condensation polaire. L'infiltrat s’étendait jusqu'au tissu musculaire squelettique sous jacent. Les annexes pilosébacées, le derme superficiel et l’épiderme étaient indemnes. On n'observait pas de prolifération néoplasique. Ces données histologiques étaient compatibles avec un diagnostic de conidiobolomycose, sans permettre de préciser l'espèce en cause. La culture ne permettait pas de préciser le phénotype. Il était traité par terbinafine 250 mg/jour pendant 1 mois sans amélioration, puis par itraconazole 300 mg/jour pendant 2 mois permettant une amélioration discrète, motivant l'adjonction d'une corticothérapie sous forme de bolus de méthylprednisone 240 mg/jour pendant 3 jours relayée per os par de la prednisone à 0,5 mg/kg soit 30 mg pendant 3 mois grâce à laquelle était obtenue une régression totale des douleurs, de la gêne respiratoire, des adénopathies, de la tuméfaction indurée du front des paupières et des pommettes, et de l'induration de la lèvre supérieure et du nez. Cependant, une tuméfaction résiduelle (Fig. 3 et 4) faisait discuter d'une prise en charge chirurgicale complémentaire, trop onéreuse pour le patient, qui secondairement était perdu de vue.

**Figure 2:**
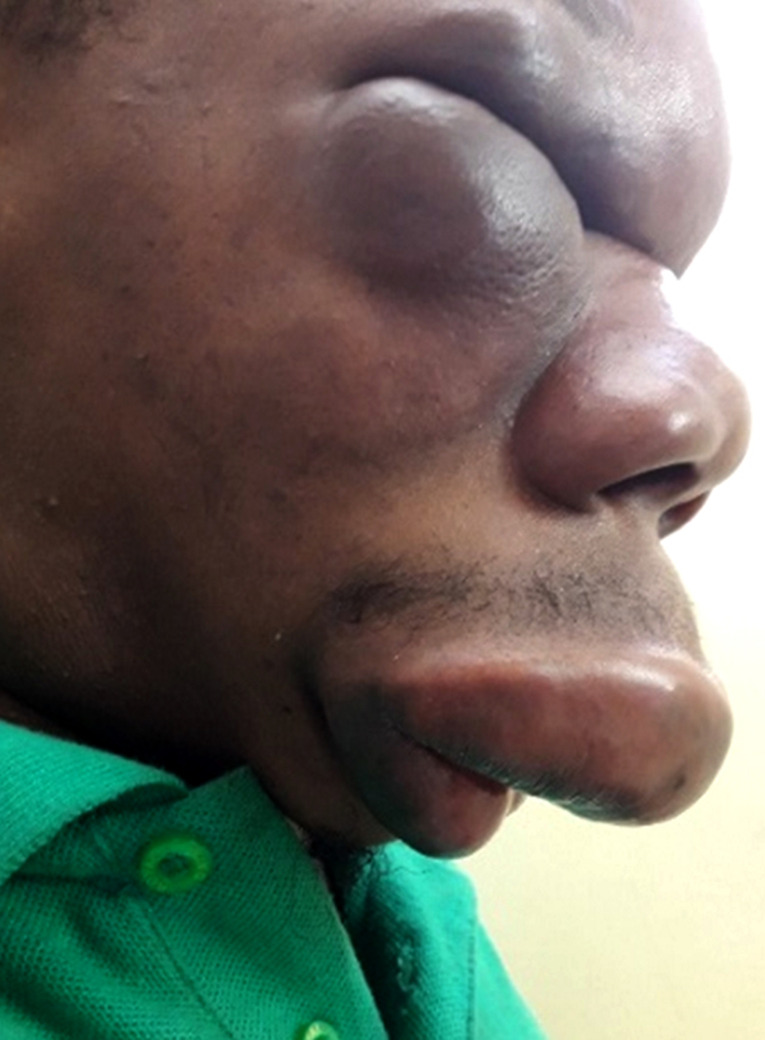
Visage du patient de profil Patient's face in profile

**Figure 3:**
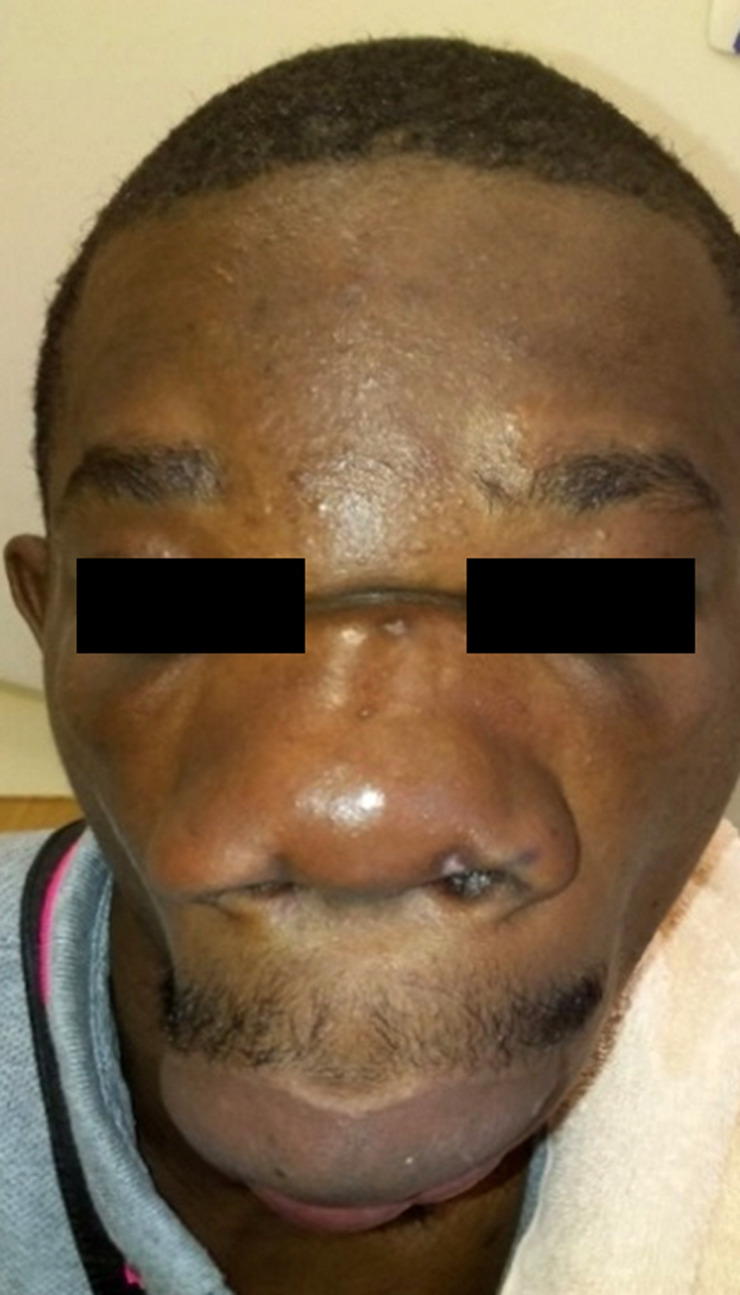
Évolution à 3 mois de traitement médical (face) Evolution after 3 months of drug treatment (front view)

**Figure 4:**
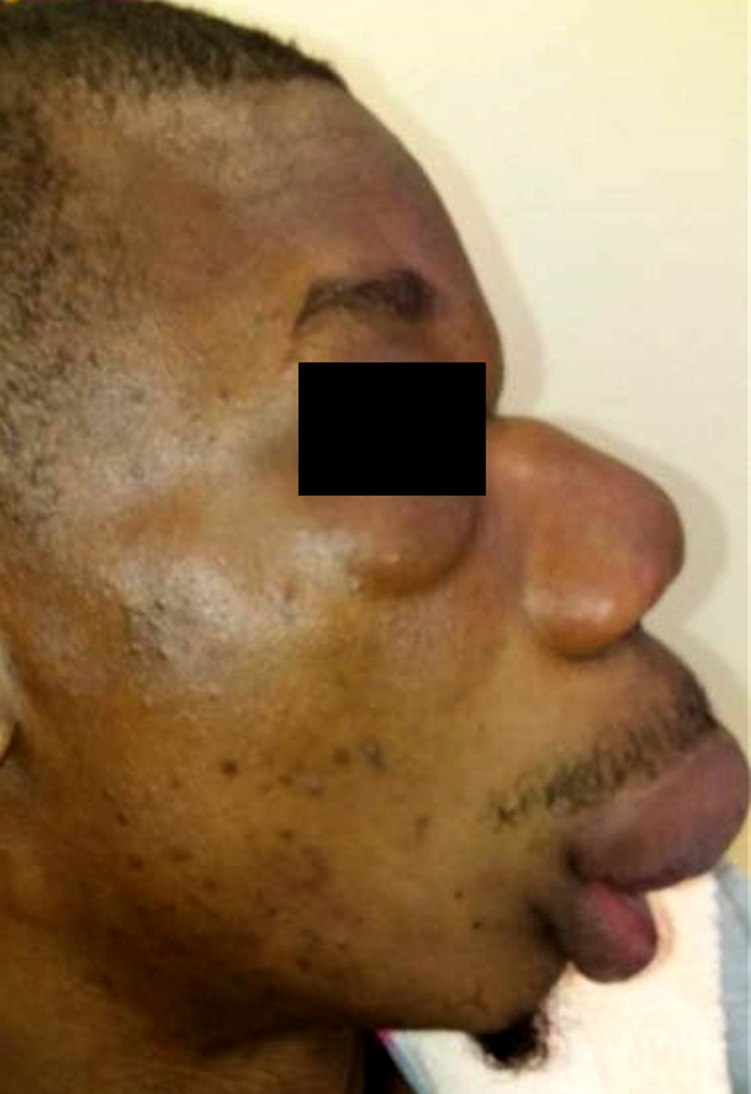
Évolution à 3 mois de traitement médical (profil) Evolution after 3 months of drug treatment (profile view)

## Discussion

Les entomophthoromycoses sont des mycoses sous-cutanées. La conidiobolomycose, dont la localisation est faciale a été rapportée pour la première fois par Blaché en 1961 chez un patient camerounais adulte [2]. À ce jour, on trouve dans la littérature une centaine de cas [3, 5] diagnostiqués exclusivement en région tropicale humide d'Afrique centrale et de l'ouest, d'Amérique, d'Asie, et des îles de l'océan Indien [13, 14]. Cette mycose est due à *Conidiobolus coronatus,* champignon saprophyte du sol, retrouvé dans l'humus et le terreau, dont la contamination se fait par inhalation de spores aérosolisées qui ensemencent les cavités sinusiennes conduisant à une déformation progressive du visage par atteinte sous-cutanée de la face et de la région naso-sinusienne, puis à un éléphantiasis du nez et de la lèvre supérieure, réalisant un aspect caricatural en museau d'hippopotame, de chien ou un visage en groin de tapir. Notre observation est la seconde rapportée au Gabon après celle de Blumentrath et al [3].

Dans ces 2 observations gabonaises, les patients vivaient dans deux villes (Lébamba, Fougamou) de la même province du Gabon (la Ngounié) – ce qui pourrait étayer l'hypothèse d'une zone d'exposition – signalaient une évolution des symptômes sur 3 et 6 mois. Ils présentaient comme dans les observations de la littérature une tuméfaction rhino-faciale caractéristique [12, 14]. En Afrique subsaharienne, la présentation clinique de la conidiobolomycose, conduit à discuter diverses pathologies: 1) infectieuses tropicales à tropisme nasal (leishmaniose, rhinosclérome, rhinosporidiose, et lèpre lépromateuse), et 2) tumorales (lymphome de Burkitt ou un lymphome NK devant l'existence d'adénopathies cervicales bilatérales) chez ce sujet jeune, et 3) un angioœdème facial. La biopsie avec analyse anatomopathologique permet de porter le diagnostic d'entomophthoromycose, la distinction entre conidiobolomycose et basidiobolomycose reposant sur la présentation clinique. L'aspect anatomopathologique est caractérisé par la présence de filaments irréguliers, larges, peu ou pas septés, entourés par un manchon de polynucléaires éosinophiles (réaction de Slendore-Hoeppli), sans envahissement vasculaire. La culture lorsqu'elle est possible doit se faire sur milieu de Sabouraud sans chloramphénicol ni cycloheximide à 30 °C. On obtient facilement des cultures dont les aspects macroscopiques et microscopiques permettent d'identifier *Conidiobolus coronatus* [11, 14]. La déformation rhinofaciale fortement suggestive/évocatrice est la conséquence d'un granulome endonasal envahissant progressivement la région du nez puis la face. L'envahissement des os du nez ou du sinus est très rare. L’évolution est en général très lente, mais a été probablement sous-estimée dans le cas de notre patient.

Sur le plan thérapeutique, les imidazolés constituent actuellement le traitement de choix [1, 4, 8, 10]. Ceux apparues comme les plus efficaces dans cette infection sont le fluconazole, le kétoconazole et l'itraconazole. Cette dernière molécule a été retenue après échec de la terbinafine initiée en première intention, et devant la toxicité hépatique reconnue du kétoconazole qui nous faisait récuser cet antifongique. L'efficacité partielle de l'itraconazole associée à la persistance de l'obstruction nasale nous ont conduit à adjoindre une corticothérapie, dont la combinaison a permis une amélioration significative et plus rapide du fait de son activité sur les remaniements inflammatoires des lésions. Dans une méta-analyse portant sur 145 patients, Blumentrath et al [3], rapportent un taux de guérison de 100% au début de la maladie, de 82% lors de maladie intermédiaire (antécédents de la maladie de moins de 12 mois), de 43 *%* lorsque les patients présentent une déformation faciale, et de 37,5% pour Choon et al [6], lors d’éléphantiasis facial. Si dans le cas de notre patient, le traitement antifongique a partiellement amélioré la dysmorphie faciale, la durée courte de ce traitement fait craindre une reprise du processus infectieux à moyen terme, que nous n'avons pu évaluer du fait de la perte de vue du patient.

Pour les deux patients gabonais, une réponse partielle était obtenue dans le premier cas sous traitement antifongique per os (terbinafine 250 mg/jour pendant 1 mois puis itraconazole 300 mg/jour) et fluconazole 400 mg/jour, terbinafine 250 mg per os pendant 9 mois dans le second cas. Une chirurgie complémentaire à but esthétique aurait pu être proposée comme cela a été rapporté dans la littérature [1, 2, 3, 6, 7] mais s'est heurtée aux limites du plateau local. Habituellement, le pronostic de cette affection est favorable [5, 9].

## Conclusion

La conidiobolomycose ou entomophthoromycose rhinofaciale est un diagnostic à évoquer en zone tropicale devant une tuméfaction indurée du haut du visage. L'association d'imidazolés à une corticothérapie précoce améliore de façon significative l'esthétique faciale du patient. Dans certains cas, le recours à une chirurgie complémentaire peut s'avérer nécessaire.

## Contribution des auteurs

Ntsame Ngoua Stéphanie: conception et rédaction

Iba Ba Josaphat: rédaction et relecture

Coniquet Sophie: relecture et collecte de données

Moussirou Soumbou Ghislaine: recherche bibliographique, relecture

Ntoutoume Sima François: analyse anatomocyto-pathologique de la biopsie cutanée

Boguikouma Jean Bruno: conception, relecture, approbation de la version finale

## Liens d'intérêts

Les auteurs ne déclarent aucun conflit d'intérêts.
